# Supplementary figures and images for: Performance of methods for SARS-CoV-2 variant detection and abundance estimation within mixed population samples
Source: PeerJ. 2023 Jan 26;11:e14596. doi: 10.7717/peerj.14596 (PMC9884472; doi:10.7717/peerj.14596)

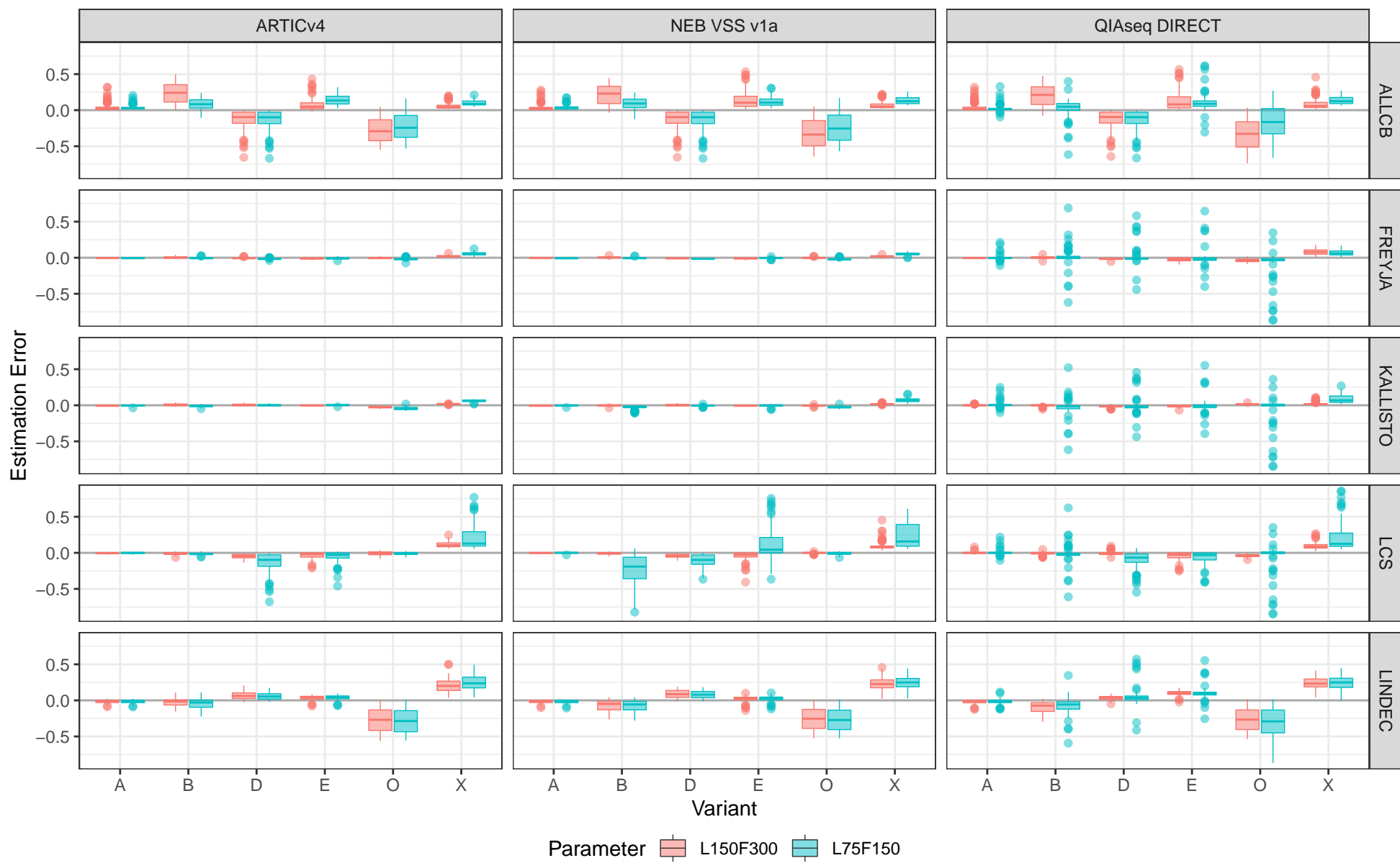

Supplement: Supplemental Information 1 — L75F150, read length of 75 and fragment size of 150; L150F300, read length of 150 and fragment size of 300; A, Alpha; B, Beta; D, Delta; E, Epsilon; O, Omicron; X, Other. [file peerj-11-14596-s001.pdf]

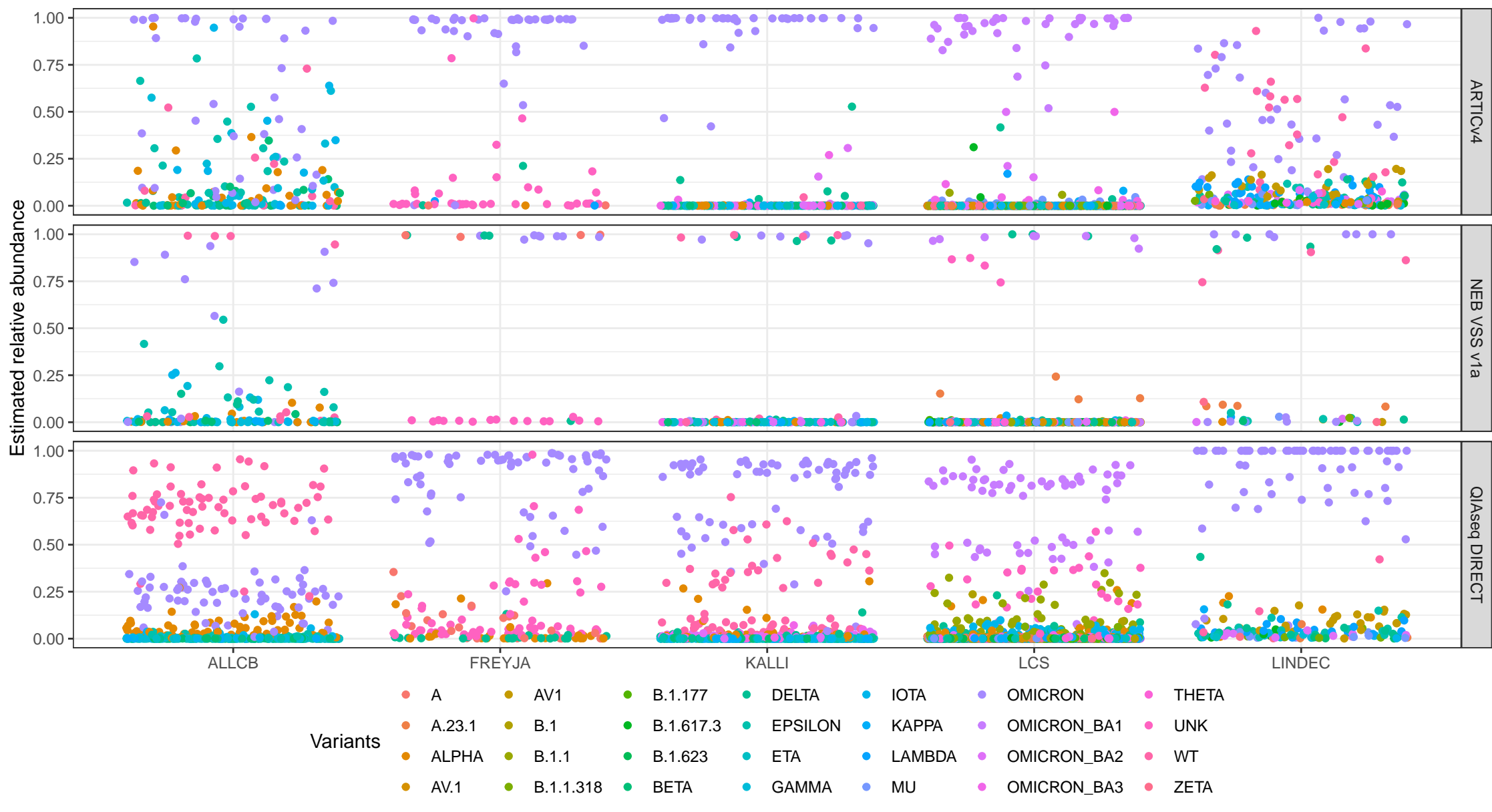

Supplement: Supplemental Information 2 [file peerj-11-14596-s002.pdf]
