# Supplementary material for: Performance of methods for SARS-CoV-2 variant detection and abundance estimation within mixed population samples
Source: PeerJ. 2023 Jan 26;11:e14596. doi: 10.7717/peerj.14596 (PMC9884472; doi:10.7717/peerj.14596)
Supplement: Supplemental Information 3 [file peerj-11-14596-s003.pdf]

Run	Enrichment Kit	Run	Enrichment Kit	Run	Enrichment Kit
SRR17978072	QIAseq DIRECT	SRR17843606	QIAseq DIRECT	SRR17771317	NEB VSS v1a
SRR17951285	QIAseq DIRECT	SRR17843607	QIAseq DIRECT	SRR17771318	NEB VSS v1a
SRR17951287	QIAseq DIRECT	SRR17843608	QIAseq DIRECT	SRR17975893	ARTICv4
SRR17951288	QIAseq DIRECT	SRR17843609	QIAseq DIRECT	SRR17975894	ARTICv4
SRR17951291	QIAseq DIRECT	SRR17843610	QIAseq DIRECT	SRR17975895	ARTICv4
SRR17951134	QIAseq DIRECT	SRR17770248	QIAseq DIRECT	SRR17975896	ARTICv4
SRR17951286	QIAseq DIRECT	SRR17843589	QIAseq DIRECT	SRR17975897	ARTICv4
SRR17951289	QIAseq DIRECT	SRR17843590	QIAseq DIRECT	SRR17975898	ARTICv4
SRR17951290	QIAseq DIRECT	SRR17786018	QIAseq DIRECT	SRR17975899	ARTICv4
SRR17951292	QIAseq DIRECT	SRR17786017	QIAseq DIRECT	SRR17975900	ARTICv4
SRR17951293	QIAseq DIRECT	SRR17786019	QIAseq DIRECT	SRR17975901	ARTICv4
SRR17839739	QIAseq DIRECT	SRR18017651	QIAseq DIRECT	SRR17975902	ARTICv4
SRR17839740	QIAseq DIRECT	SRR18017654	QIAseq DIRECT	SRR17975903	ARTICv4
SRR17839741	QIAseq DIRECT	SRR18017730	QIAseq DIRECT	SRR17975904	ARTICv4
SRR17839742	QIAseq DIRECT	SRR18017789	QIAseq DIRECT	SRR17975905	ARTICv4
SRR17839744	QIAseq DIRECT	SRR18018920	QIAseq DIRECT	SRR17975906	ARTICv4
SRR17839753	QIAseq DIRECT	SRR18017727	QIAseq DIRECT	SRR17975907	ARTICv4
SRR17839756	QIAseq DIRECT	SRR18017658	QIAseq DIRECT	SRR17975908	ARTICv4
SRR17839757	QIAseq DIRECT	SRR18017669	QIAseq DIRECT	SRR17975909	ARTICv4
SRR17839758	QIAseq DIRECT	SRR18017674	QIAseq DIRECT	SRR17975910	ARTICv4
SRR17839755	QIAseq DIRECT	SRR18017683	QIAseq DIRECT	SRR17975911	ARTICv4
SRR17839754	QIAseq DIRECT	SRR18017686	QIAseq DIRECT	SRR17975912	ARTICv4
SRR17839761	QIAseq DIRECT	SRR18017687	QIAseq DIRECT	SRR17975913	ARTICv4
SRR17691854	QIAseq DIRECT	SRR18017700	QIAseq DIRECT	SRR17975914	ARTICv4
SRR17691855	QIAseq DIRECT	SRR18017717	QIAseq DIRECT	SRR17975915	ARTICv4
SRR17691856	QIAseq DIRECT	SRR18017724	QIAseq DIRECT	SRR17975916	ARTICv4
SRR17691857	QIAseq DIRECT	SRR18017791	QIAseq DIRECT	SRR17975917	ARTICv4
SRR17540870	QIAseq DIRECT	SRR18017794	QIAseq DIRECT	SRR17975918	ARTICv4
SRR17540872	QIAseq DIRECT	SRR17720373	NEB VSS v1a	SRR17975919	ARTICv4
SRR17540873	QIAseq DIRECT	SRR17720374	NEB VSS v1a	SRR17975920	ARTICv4
SRR17540875	QIAseq DIRECT	SRR17720375	NEB VSS v1a	SRR17975921	ARTICv4
SRR17540877	QIAseq DIRECT	SRR17720376	NEB VSS v1a	SRR17975922	ARTICv4
SRR17540871	QIAseq DIRECT	SRR17721366	NEB VSS v1a	SRR17975923	ARTICv4
SRR17540874	QIAseq DIRECT	SRR17721367	NEB VSS v1a	SRR17975924	ARTICv4
SRR17540876	QIAseq DIRECT	SRR17721368	NEB VSS v1a	SRR17975925	ARTICv4
SRR17843586	QIAseq DIRECT	SRR17721369	NEB VSS v1a	SRR17975926	ARTICv4
SRR17843587	QIAseq DIRECT	SRR17066048	NEB VSS v1a	SRR17975927	ARTICv4
SRR17843588	QIAseq DIRECT	SRR17066051	NEB VSS v1a	SRR17975928	ARTICv4
SRR17843591	QIAseq DIRECT	SRR17066052	NEB VSS v1a	SRR17975929	ARTICv4
SRR17843592	QIAseq DIRECT	SRR17771315	NEB VSS v1a	SRR17975930	ARTICv4
SRR17843605	QIAseq DIRECT	SRR17771316	NEB VSS v1a	SRR17975931	ARTICv4

Table S1. SRA accession numbers of empirical wastewater samples used in this study.
