# Supplementary material for: Performance of methods for SARS-CoV-2 variant detection and abundance estimation within mixed population samples
Source: PeerJ. 2023 Jan 26;11:e14596. doi: 10.7717/peerj.14596 (PMC9884472; doi:10.7717/peerj.14596)
Supplement: Supplemental Information 4 — ACTUAL, known relative abundance of variants in simulated data; L75F150, single end read length of 75 and simulated total fragment size of 150; L150F300, read length of 150 and fragment size of 300. [file peerj-11-14596-s004.pdf]

Method 1	Method 2	Configuration	ARTICv4	NEB VSS v1a	QIAseq DIRECT
ACTUAL	ALLCB	L75F150	0.4924 (0.4324, 0.5481)	0.4600 (0.4018, 0.5144)	0.4685 (0.3917, 0.5389)
ACTUAL	ALLCB	L150F300	0.4713 (0.4064, 0.5314)	0.4014 (0.3417, 0.4578)	0.3971 (0.3390, 0.4522)
ACTUAL	FREYJA	L75F150	0.9855 (0.9816, 0.9886)	0.9880 (0.9848, 0.9905)	0.7817 (0.6579, 0.8643)
ACTUAL	FREYJA	L150F300	0.9966 (0.9958, 0.9972)	0.9971 (0.9965, 0.9977)	0.9658 (0.9565, 0.9731)
ACTUAL	KALLISTO	L75F150	0.9794 (0.9750, 0.9831)	0.9725 (0.9671, 0.9770)	0.7714 (0.6483, 0.8552)
ACTUAL	KALLISTO	L150F300	0.9952 (0.9940, 0.9962)	0.9976 (0.9971, 0.9979)	0.9929 (0.9911, 0.9944)
ACTUAL	LCS	L75F150	0.6807 (0.5980, 0.7491)	0.4084 (0.3405, 0.4721)	0.5911 (0.4776, 0.6851)
ACTUAL	LCS	L150F300	0.9295 (0.9109, 0.9443)	0.9382 (0.9070, 0.9592)	0.9408 (0.9234, 0.9543)
ACTUAL	LINDEC	L75F150	0.5307 (0.4740, 0.5830)	0.5346 (0.4848, 0.5810)	0.4301 (0.3658, 0.4904)
ACTUAL	LINDEC	L150F300	0.5719 (0.5126, 0.6257)	0.5590 (0.5067, 0.6071)	0.5158 (0.4659, 0.5624)
ALLCB	FREYJA	L75F150	0.5143 (0.4541, 0.5699)	0.4879 (0.4270, 0.5445)	0.5978 (0.5255, 0.6615)
ALLCB	FREYJA	L150F300	0.4766 (0.4098, 0.5384)	0.4064 (0.3463, 0.4632)	0.4022 (0.3431, 0.4581)
ALLCB	KALLISTO	L75F150	0.5233 (0.4619, 0.5797)	0.4670 (0.4056, 0.5241)	0.5849 (0.5140, 0.6478)
ALLCB	KALLISTO	L150F300	0.4914 (0.4266, 0.5512)	0.3984 (0.3397, 0.4540)	0.3849 (0.3280, 0.4390)
ALLCB	LCS	L75F150	0.4438 (0.3803, 0.5031)	0.1136 (0.0711, 0.1556)	0.4890 (0.4125, 0.5587)
ALLCB	LCS	L150F300	0.4536 (0.3864, 0.5161)	0.3654 (0.3063, 0.4216)	0.3708 (0.3138, 0.4251)
ALLCB	LINDEC	L75F150	0.4505 (0.3828, 0.5134)	0.3998 (0.3319, 0.4636)	0.5011 (0.4161, 0.5775)
ALLCB	LINDEC	L150F300	0.4186 (0.3541, 0.4792)	0.3228 (0.2696, 0.3739)	0.3625 (0.3080, 0.4146)
FREYJA	KALLISTO	L75F150	0.9928 (0.9913, 0.9941)	0.9902 (0.9872, 0.9925)	0.9752 (0.9687, 0.9805)
FREYJA	KALLISTO	L150F300	0.9962 (0.9951, 0.9970)	0.9981 (0.9977, 0.9985)	0.9736 (0.9659, 0.9796)
FREYJA	LCS	L75F150	0.7485 (0.6673, 0.8122)	0.4376 (0.3624, 0.5073)	0.7701 (0.6926, 0.8301)
FREYJA	LCS	L150F300	0.9467 (0.9332, 0.9576)	0.9540 (0.9262, 0.9715)	0.9834 (0.9776, 0.9878)
FREYJA	LINDEC	L75F150	0.5660 (0.5072, 0.6195)	0.5741 (0.5229, 0.6211)	0.6202 (0.5593, 0.6745)
FREYJA	LINDEC	L150F300	0.5838 (0.5235, 0.6383)	0.5689 (0.5161, 0.6175)	0.5585 (0.5089, 0.6045)
KALLISTO	LCS	L75F150	0.7198 (0.6364, 0.7866)	0.4666 (0.3869, 0.5395)	0.8066 (0.7377, 0.8588)
KALLISTO	LCS	L150F300	0.9351 (0.9169, 0.9494)	0.9475 (0.9178, 0.9666)	0.9544 (0.9404, 0.9651)
KALLISTO	LINDEC	L75F150	0.6050 (0.5461, 0.6580)	0.5882 (0.5341, 0.6376)	0.5843 (0.5180, 0.6435)
KALLISTO	LINDEC	L150F300	0.6103 (0.5507, 0.6638)	0.5806 (0.5280, 0.6288)	0.5024 (0.4519, 0.5496)
LCS	LINDEC	L75F150	0.3379 (0.2740, 0.3988)	0.2012 (0.1633, 0.2386)	0.3778 (0.3187, 0.4340)
LCS	LINDEC	L150F300	0.5968 (0.5353, 0.6520)	0.5528 (0.5025, 0.5993)	0.5683 (0.5186, 0.6143)

Table S2. Concordance correlation coefficient (CCC) with bootstrap 95% lower and upper confidence interval limits between methods and actual relative abundances on simulated data for all possible combinations of amplicon panel and read/fragment length used. ACTUAL = known relative abundance of variants in simulated data; L75F150=single end read length of 75 and simulated total fragment size of 150; L150F300=read length of 150 and fragment size of 300.
